# Determination of 3′-Sialyllactose in Edible Bird’s Nests and the Effect of Stewing Conditions on the 3′-Sialyllactose Content of Edible Bird’s Nest Products

**DOI:** 10.3390/molecules28041703

**Published:** 2023-02-10

**Authors:** Xiaojiang Zhang, Ruifang Liang, Weijuan Bai, Yue’e Xiao, Xuncai Liu, Qunyan Fan, Baozhong Guo

**Affiliations:** Research Institute of Bird’s Nest, Xiamen Yan Palace Seelong Food Co., Ltd., Xiangming Road 3, Xiamen 361100, China

**Keywords:** 3′-sialyllactose, high-performance liquid chromatography-tandem mass spectrometry, edible bird’s nest

## Abstract

Sialyllactose is an acidic oligosaccharide that has an immune-protective effect against pathogens and contributes to developing the immune system and intestinal microbes. In this study, a method for the determination of 3′-sialyllactose by high-performance liquid chromatography tandem mass spectrometry was established. The sample was treated with 0.1% formic acid methanol solution, and the gradient elution was performed with 0.05% formic acid water and 0.1% formic acid acetonitrile. The hydrophilic liquid chromatographic column was used for separation. The results showed that the linearity was good in the concentration range of 1~160 μg/L. The limit of detection (LOD) and the limit of quantification (LOQ) of the method were 0.3 μg/kg and 1.0 μg/kg, the recovery range was 91.6%~98.4%, and the relative standard deviation (RSD) was 1.5%~2.2%. This method is fast and sensitive. In addition, the 3′-sialyllactose content in edible bird’s nest products produced by different processes was studied. It was found that within the tested range, 3′-sialyllactose in edible bird’s nest products increased with the intensity of stewing and increased with the addition of sugar. In short, the results provided a new method for detecting the nutritional value of edible bird’s nests, as well as a new direction for improving the nutritional value of edible bird’s nest products.

## 1. Introduction

The edible bird’s nest is mainly produced in Southeast Asia and has rich nutritional and medicinal value. Edible bird’s nest is a natural food made from the saliva of the swifts of *Aerodromus* and *Collasalia*. The main consumer group is Asians. It is believed to improve overall health. As early as the Tang Dynasty, edible bird’s nest was considered a high-end health product and status symbol. Modern studies have shown that the main nutritional components of edible bird’s nest contain protein (62%~63%), carbohydrate (25.62%~27.26%), sialic acid (10%), and trace elements (including calcium, magnesium, sodium, and potassium) [1,2]. The composition of natural bird’s nests may vary due to the influence of season, foraging, species, environmental factors, and geographical location, such as nitrite, total arsenic, and lead. However, numerous studies have shown that there was no significant difference in the content of sialic acid in the edible bird’s nests of Malaysia and Indonesia (*p* > 0.05), which showed that the content of sialic acid in edible bird’s nests was relatively stable.

Sialic acid, as one of the most valuable components in the edible bird’s nest, usually exists as oligosaccharides, glycolipids, or glycoproteins in the edible bird’s nest [3]. Sialyllactose is a form of sialic acid storage, which is formed by the connection between sialic acid and lactose through glycosidic bonds. The reduced end of sialyllactose is a lactose group, which connects with a sialic acid residue through α-2, 3 or α-2, 6 bonds at the non-reducing end of lactose residues, forming two existing forms of 3′-sialyllactose and 6′-sialyllactose [4]. Among them, 3′-sialyllactose can act on the gut–brain axis, regulate brain metabolism, relieve nervous tension caused by stimulation, and act as a specific pathogen and toxin binding site, significantly reducing the adhesion of harmful substances and endothelial cells and improving human immunity. Moreover, it has the functions of antibacterial, anti-inflammation, protecting the infant intestine, promoting infant brain maturation, and improving learning ability [5,6,7,8]. In contrast, 6′-sialyllactose does not have these biological functions. In recent years, more and more attention has been paid to the efficacy of 3′-sialyllactose, which can be prepared by artificial synthesis. The main methods include chemical synthesis and biological synthesis. Among them, the chemical synthesis method mainly takes sialic acid as the donor and binds to the oligosaccharide receptor regioselective or stereoselective. Due to the electronic and spatial blocking effects, it is difficult to form glycosidic bonds between the two, resulting in low production efficiency and high reaction requirements. Enzymatic synthesis needs to consider the selection of enzymes, as well as the separation and purification. Both methods have certain difficulties in batch production. It can be seen that obtaining sialyllactose from natural food is the simplest way at present.

However, in the current research, although the importance of sialyllactose has been recognized by researchers, there is no mature detection method for sialyllactose in edible bird’s nests. Due to the lack of strong chromophores in sialyllactose and the low sensitivity of UV detectors, some methods rely on UV detectors, such as high-performance liquid chromatography (HPLC) [9,10], high performance anion exchange chromatography (HPAEC) [11,12], porous graphitized carbon chromatography (PGC) [13,14], and capillary electrophoresis (CE) [15,16]. Generally, it is necessary to derivate lactose sialylate so that the derivative has a certain ultraviolet absorption peak before it can be detected normally. Such methods have complicated conditions and high conditions for sample preparation and are not suitable for detecting sialyllactose [17,18,19,20,21].

Compared with common detectors, when mass spectrometry is used as a detector, it can conduct qualitative and quantitative analysis of samples faster and more accurately according to the relative molecular weight and sub-ion information and the mass spectrum corresponding to sub-ion. At present, liquid chromatography–mass spectrometry has been applied to the detection of sialyllactose. However, it is mainly applied to determining dairy products or endogenous sialyllactose. It has not been applied to the detection of sialyllactose in edible bird’s nests. Therefore, referring to the existing determination methods, liquid chromatography–mass spectrometry can potentially solve the problem that sialyllactose in edible bird’s nests cannot be completely, qualitatively, and quantitatively separated in liquid phase detection.

According to the current report [22,23], the pretreatment of dairy products generally adopts the reduction method; that is, the sample is centrifuged to remove fat and protein, then reduced with NaBH4, eluted by graphite carbon column, dried in a vacuum, finally dissolved in deionized water, and detected by liquid chromatography–mass spectrometry. This treatment method is complex, and the elution process is time-consuming. Jang et al. [19] used high-performance liquid chromatography–mass spectrometry to determine the content of salivary lactose in rat plasma, optimized the pretreatment process, selected methanol for protein precipitation in rat plasma samples, completely evaporated the methanol extract with a vacuum concentrator, and re-dissolved with a solution containing 10 mM ammonium acetate (pH 4.5) and acetonitrile (40:60, *v*/*v*). It was shown that although the determination of sialyllactose was carried out by HPLC-MS, different sample matrices led to great differences in the detection methods, including sample pretreatment, equipment conditions, and method optimization results. As shown in Table 1, the differences in the determination of sialyllactose by high-performance liquid chromatography–mass spectrometry were described in terms of sample type, pretreatment method, LOD, RSD, and rate of recovery. Compared with other literature methods, this research method used 0.1% formic acid and methanol for protein precipitation, vacuum concentration, and drying, and finally dissolved the samples in deionized water for detection by liquid chromatography–mass spectrometry. This method only used methanol for alcohol precipitation and formic acid to adjust the pH value of the sample and did not use other highly toxic chemicals, which is more friendly to the environment and is easier to operate with good precision and accuracy.

This study used ultra-high-performance liquid chromatography–mass spectrometry to establish a detection method for 3′-sialyllactose. The method is sensitive, simple in sample preparation, does not require complex pretreatment, and is not affected by complex matrices. 3′-sialyllactose contents of edible bird’s nests were accurately determined. Currently, the product forms of edible bird’s nests mainly include dry-edible bird’s nests and ready-to-eat edible bird’s nest products. The dry-edible bird’s nest requires consumers to stew it in water at 100 °C for 30 min and then cool it to room temperature according to the household stewing method. The ready-to-eat edible bird’s nest products are processed by businesses through a number of types of edible bird’s nest processing technology, which solved the problem of the bird’s nest being ready to eat and added sugar to adjust the taste of the products. In order to investigate the difference in the content of 3′-sialyllactose in edible bird’s nest products under different heat treatment conditions and the effect of sugar on the content of 3′-sialyllactose, and to explore the effect of heat treatment intensity on the nutritional value of edible bird’s nest products, the corresponding factors were investigated based on this research method.

## 2. Results and Discussion

### 2.1. Condition Optimization

#### 2.1.1. Selection of Ions for Mass Spectrometry

**Figure 1:**
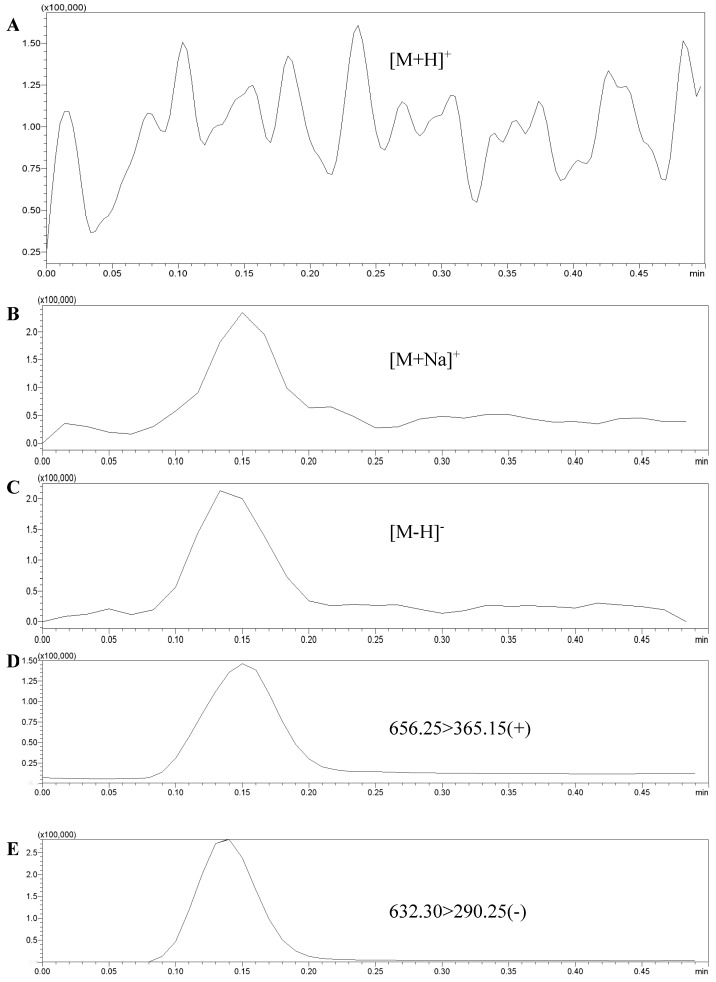
The responses of [M + Na]^+^ and [M − H]^−^ molecular ion peaks and fragment ion peaks. (**A**): [M + H]^+^ molecular ion peaks; (**B**): [M + Na]^+^ molecular ion peak; (**C**): [M − H]^−^ molecular ion peak; (**D**): [M + Na]^+^ fragment ion peak; (**E**): [M − H]^−^ fragment ion peak.

The 3′-sialyllactose standard solution (1 mg/L) was used to test and optimize the mass spectrometry conditions. After entering the ion source, the standard solution was scanned by positive ion and negative ion, respectively, and the responses of [M + Na]^+^, [M + H]^+^, and [M − H]^−^ molecular ion peaks were compared. As shown in Figure 1, there was almost no response of the [M + H]^+^ peak, and there was no significant difference between the response value of the [M + Na]^+^ peak and the [M − H]^−^ peak. However, in the cleavage of second-order mass spectrometry, it was discovered that the response of [M − H]^−^ fragment ion peak (270,000) was greater than that of [M + Na]^+^ fragment ion peak (140,000). When adding edible bird’s nest samples, there was no corresponding ion interference.

**Figure 2:**
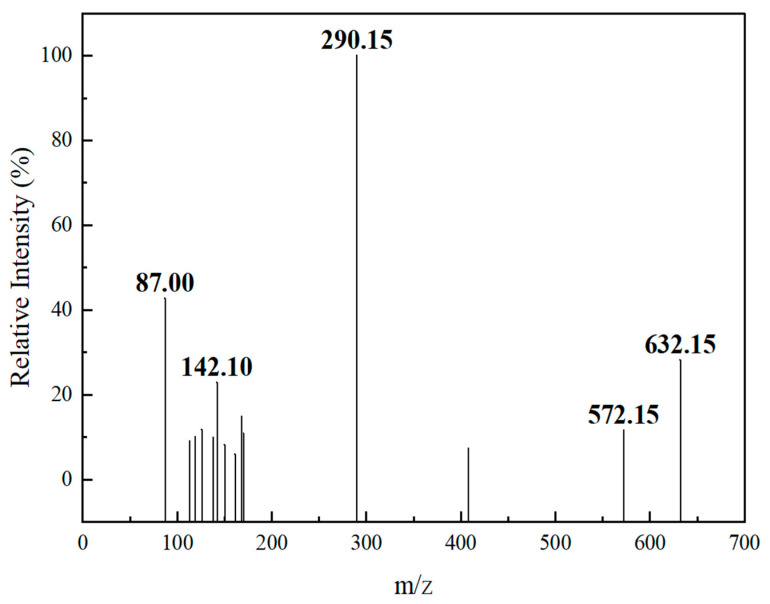
Product-ion scan spectra of 3′-sialyllactose.

Therefore, in order to improve the sensitivity of the method, in this study, the [M − H]^−^ peak was selected as the parent ion. It was bombarded with certain energy (CE) to obtain the corresponding ion fragments. Finally, two fragment ions with strong signals were selected, forming two pairs of monitoring ions with the parent ion. The optimized MRM ion pairs and mass spectrometry conditions were shown in Figure 2 and Table 2.

#### 2.1.2. Chromatographic Condition Optimization

**Figure 3:**
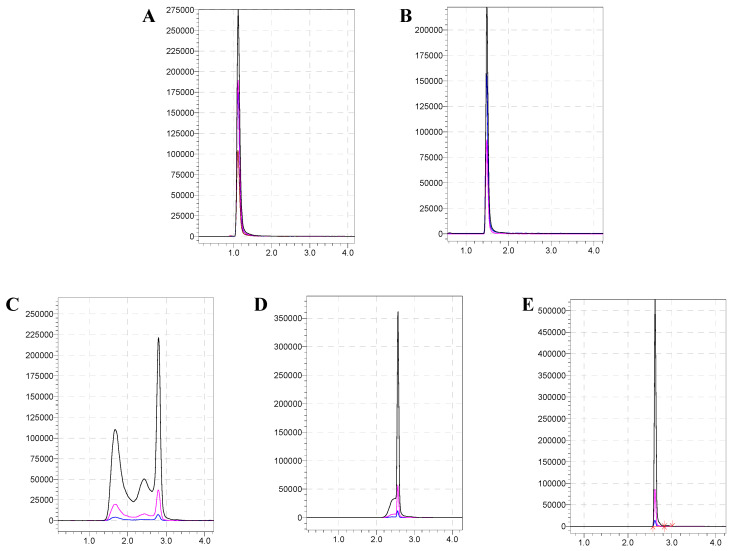
Effect of initial ratio of different mobile phases on chromatographic peak pattern. (**A**): C18 column; (**B**): C18-AQ column; (**C**–**E**): Chromatograms of HILIC columns with different initial mobile phase ratios of 75%, 83%, and 90%, respectively.

In this study, the C18, C18-AQ, and HILIC columns were compared. In the retention effect, HILIC column (2.66 min) > C18-AQ (1.56 min) > C18 (1.15 min), i.e., the HILIC column was better than other reversed-p27hase columns in sialyllactose separation. At the same time, the initial proportion of the mobile phase was further investigated, and the chromatographic separation effect was optimized. The result showed that when the initial mobile phase ratio was adjusted to 90% (mobile phase B), the poor peak shape, cross peak, or micro-tailing phenomenon could be effectively solved (as Figure 3). This is because sialyllactose is a highly polar compound, and the general reversed-phase column cannot retain it very well [24,25]. In conclusion, when the HILIC chromatographic column was used as the separation column, the test and analysis time was 5 min, the running time was 13 min, the retention time was 2.66 ± 0.05 min, the peak shape was sharp and symmetrical, and there was no tailing phenomenon, which was the optimal separation condition.

### 2.2. Method Verification

#### 2.2.1. Detection Limit, Quantitative Limit, and Linear Range of the Method

**Figure 4:**
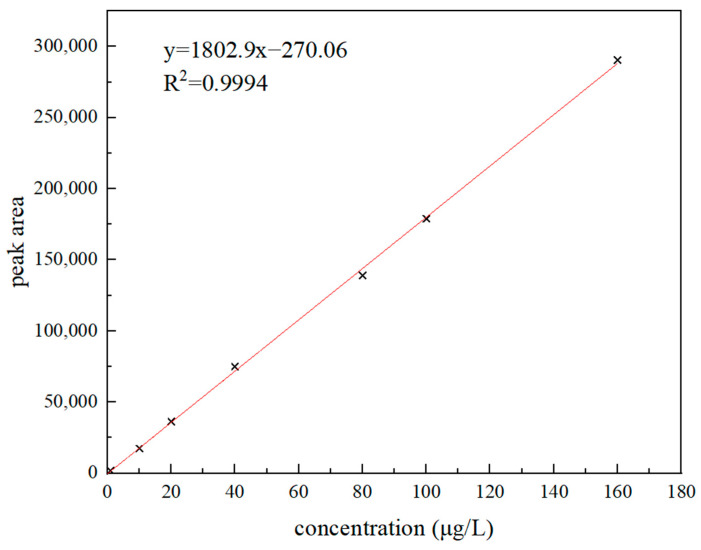
3′-sialyllactose concentration and peak area curve.

For preparing a series of 3′-sialyllactose standard solutions of 0~160 μg/L, 0 mL, 0.01 mL, 0.02 mL, 0.04 mL, 0.08 mL, 0.10 mL, and 0.16 mL of 1 mg/L standard solution were taken, respectively, and diluted to 1 mL. An amount of 0.10 mL 10 μg/L standard solution was taken and diluted to 1 mL. The standard curve regression equation was obtained. It is shown in Figure 4 that when the sample concentration range was 1~160 μg/L, the curve fitting of sample concentration and peak area was R^2^ = 0.9994, which showed that there was a good positive linear correlation. By reducing the concentration of the standard in the blank sample step by step, the detection limit (LOD) and quantitative limit (LOQ) of the method were determined. In the result, the target concentration corresponding to signal-to-noise ratio S/N ≥ 3 and S/N ≥ 10 were used as the detection limit and quantitative limit of this method. The LOD was 0.3 μg/kg, and the LOQ was 1.0 μg/kg.

#### 2.2.2. Precision and Accuracy of the Method

The edible bird’s nest sample was measured in parallel three times. The average value was 112.5 μg/kg, which was used as the background value of the sample. Then, 0.10 mL, 0.20 mL, and 0.40 mL of intermediate standard solutions with a concentration of 1 mg/L were taken and added to the edible bird’s nest samples, respectively, i.e., the additional amounts were 50 μg/kg, 100 μg/kg, and 200 μg/kg, respectively. Then, pretreatment and determination (as described in Section 3.2) were carried out, and their concentrations were within the concentration range of the standard curve.

Through the addition recovery test, the accuracy and precision of the method were investigated. The results were shown in Table 3. The results showed that the average recovery range of 3′-sialyllactose was 91.6%~98.4%, and the relative standard deviation (RSD) was 1.5%~2.2%. According to the standard of China (GB/T 27417-2017) [26], the accuracy and precision of the method determined by the test meeting the relevant requirements, i.e., the method is reliable.

### 2.3. Effect of Heat Treatment Intensity on 3′-Sialyllactose Content of Edible Bird’s Nest Products

#### 2.3.1. Effect of Heat Treatment Temperature on 3′-Sialyllactose of Edible Bird’s Nest Product

**Figure 5:**
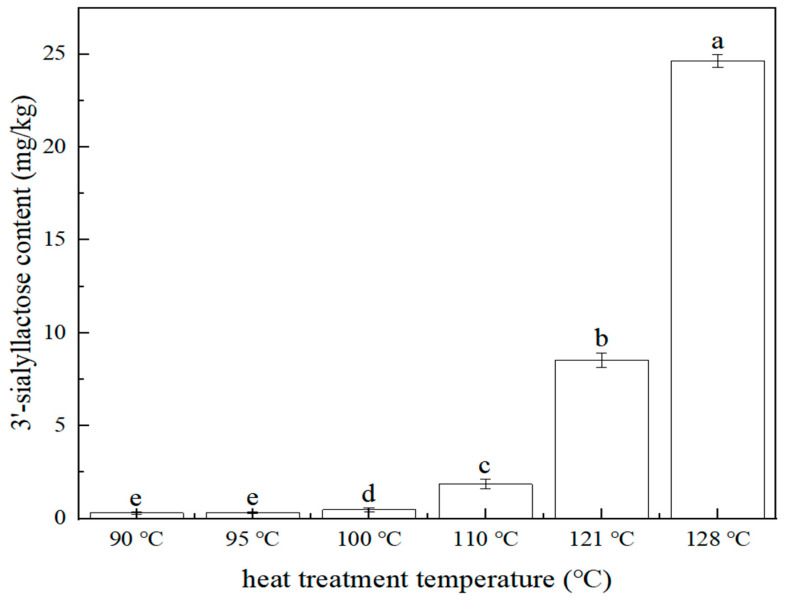
Relationship between 3′-sialyllactose content and heat treatment temperature (*n* = 2, *p* < 0.05). The same letter means no significant difference at the 0.05 level, while different letters mean significant difference at the 0.05 level. The average value of the largest group is labeled as (a), and the groups with significant differences increase alphabetically according to the average size, as below.

The effect of heat treatment (stewing) temperature (90 °C, 95 °C, 100 °C, 115 °C, 121 °C, 128 °C) on the 3′-sialyllactose content of the edible bird’s nest product was investigated. The sample preparation is referred to in Section 3.3.1. The content of 3′-sialyllactose at different heat treatment temperatures is shown in Figure 5. The result showed that with the increased heat treatment temperature, the content of 3′-sialyllactose in edible bird’s nest product increased. When the temperature was lower than 95 °C, the content of 3′-sialyllactose was very low, but when the temperature was higher than 100 °C, 3′-sialyllactose increased significantly (*p* < 0.05). Therefore, it was indicated that within the tested range, the increased stewing temperature in the production of edible bird’s nest products was beneficial to the transformation and formation of 3′-sialyllactose.

#### 2.3.2. Effect of Heat Treatment Time on 3′-Sialyllactose of Edible bird’s Nest Products

**Figure 6:**
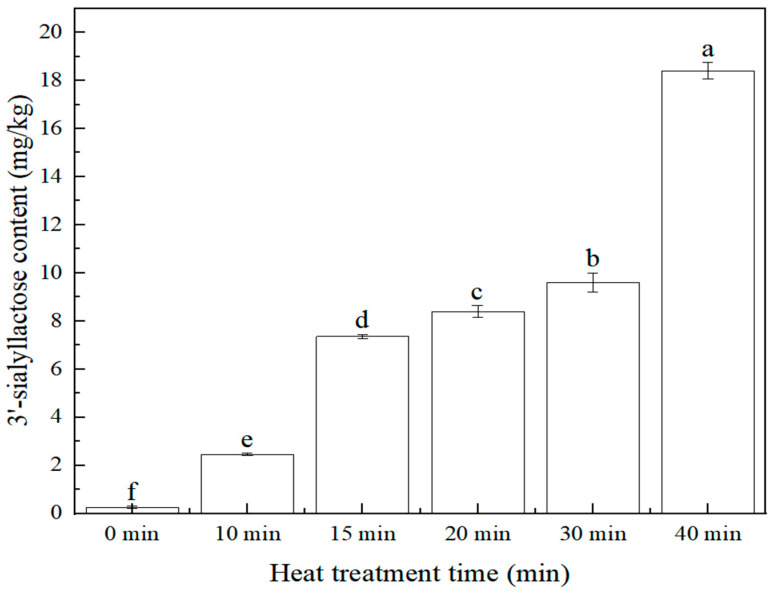
Relationship between 3′-sialyllactose content and heat treatment time (*n* = 2, *p* < 0.05).

The effect of heat treatment (stewing) time (0 min, 10 min, 15 min, 20 min, 30 min, 40 min) on the 3′-sialyllactose content of the edible bird’s nest product was investigated. The sample preparation is referred to in Section 3.3.2. The content of 3′-sialyllactose at different heat treatment times is shown in Figure 6. The result showed that with the extended heat treatment time, the content of 3′-sialyllactose in edible bird’s nest products increased. When the heat treatment time was 15~30 min, the content of 3′-sialyllactose was relatively stable, and the range was 7.35~9.60 mg/kg. With the extension of heat treatment time, the 3′-sialyllactose content in edible bird’s nest products increased continuously. Therefore, it was indicated that within the tested range, the increased stewing time in the production of edible bird’s nest products was beneficial to the transformation and formation of 3′-sialyllactose.

In summary, the heat treatment intensity (temperature and time) could affect the change of 3′-sialyllactose. Within the tested range, the stronger the heat treatment intensity, the higher the 3′-sialyllactose content. According to Yagi [27], most of the sialic acid in edible bird’s nests is bound by $\mathrm{Sialic}\;\mathrm{acid}\overset{\alpha -2,3\;or\;\alpha -2,6}{\to }Gal\overset{\beta -1,3\;\mathrm{or}\;\beta -1,4}{\to }GlcNAC\to \mathrm{polysaccharide}\;\mathrm{chain}$ to protein. Therefore, it was speculated that the increase in heat treatment intensity accelerated the hydrolysis of $Gal\overset{\beta -1,3\;\mathrm{or}\;\beta -1,4}{\to }GlcNAC$ and promoted the transformation and formation of 3′-sialyllactose.

### 2.4. Effect of Sugar on 3′-Sialyllactose of Edible Bird’s Nest Producst

**Figure 7:**
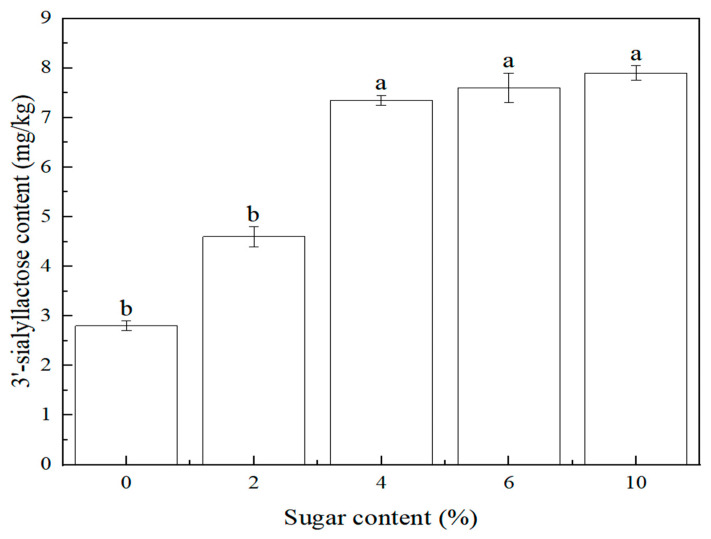
Relationship between 3′-sialyllactose content and sugar content (*n* = 2, *p* < 0.05).

At present, ready-to-eat edible bird’s nest products on the market can be divided into two categories: sugar-added and no-sugar-added. In addition, the sugar content is basically in the range of 0~10%. During the research process, it was found that the content of sugar would affect the detection of sialyllactose, so it was listed as an investigation factor. This study investigated the effect of sugar content (0%, 2%, 4%, 6%, 10%) on the 3′-sialyllactose content of the edible bird’s nest product. The sample preparation is referred to in 3.4. The content of 3′-sialyllactose at different sugar contents is shown in Figure 7. The result showed that with the increased sugar content, the content of 3′-sialyllactose in edible bird’s nest products increased. Under the same conditions, the content of 3′-sialyllactose in the sugar-added samples was four times higher than that in the unsugar-added sample. When the amount of added sugar was more than 4%, 3′-sialyllactose reached a relative balance. Therefore, the result indicated that sugar within a certain concentration range could promote the transformation and formation of 3′-sialyllactose.

The oligosaccharide content change caused by adding sugar during processing has not yet been explained. From this study, it was speculated that monocrystal rock sugar (used in this study) could be hydrolyzed into glucose and fructose [28,29,30,31], in which the glucose could be dehydrated and condensed with galactose sialate by heat treatment to form 3′-sialyllactose. In sum, it showed that adding sugar (monocrystal rock sugar) could increase the nutritional value of edible bird’s nest products within the tested range.

### 2.5. Application Examples of the Detection Method

In order to investigate the 3′-sialyllactose content in actual edible bird’s nest products, the method established in this study was used to determine the 3′-sialyllactose content of nine kinds of products sold in the market. The sample types were dry-edible bird’s nests, bottle-packed ready-to-eat edible bird’s nest products, and bowl-packed ready-to-eat edible bird’s nest products. Each sample was measured twice, and the average value was taken, as shown in Table 4. Among them, sample 1 was a dry-edible bird’s nest, and the stewing conditions was 100 °C in water for 30 min and then colling to room temperature, simulating the method of home stewing. In addition, all samples were treated according to 3.2.1 before detection.

As shown in Table 5, there were differences in 3′-sialyllactose content in different styles of edible bird’s nest products. The average content of ready-to-eat edible bird’s nest products (183.5 μg/kg) was higher than the home-stewing dry-edible bird’s nest (6.60 μg/kg); that is, the detected amount of 3′-sialyllactose of ready-to-eat edible bird’s nest products was over 30 times that of 100 °C home-stewing dry-edible bird’s nest. Among them, ready-to-eat edible bird’s nest products were processed by merchants with a certain heat treatment intensity, stronger than home-stewing dry-edible bird’s nest, which might be the main reason for their higher 3′-sialyllactose content. Therefore, it was indicated that higher heat treatment intensity could improve the nutritional value of edible bird’s nest products to a certain extent.

## 3. Materials and Methods

### 3.1. Materials

Edible bird’s nest (made from house nest in Indonesia, *Apodidiae*) was provided by Xiamen Yan Palace Seelong Food Co., Ltd. Edible bird’s nest products (Sample-1, Sample-2, Sample-3, Sample-4, Sample-5, Sample-6, Sample-7, Sample-8, Sample-9, made from house nest in Indonesia, *Apodidiae*) were commercially available edible bird’s nest products. 3′-Sialyllactose (purity 99%) was provided by Xiamen University. Methanol and formic acid were used for chromatographic purity, provided by TEDIA. Other reagents used were of analytical grade and above.

### 3.2. Optimization of Determination Methods and Conditions

#### 3.2.1. Pretreatment Condition

After homogenization, 2.00 g (±0.1 g) of the uniform sample (edible bird’s nest) was mixed with 0.1% formic acid methanol solution to fix the volume to 10 mL. After reacting for 10 min, the sample was centrifuged by 10,000 r/min for 10 min. The 5 mL of the separated supernatant was placed in a nitrogen blow tube to be nearly dried. Subsequently, the volume was fixed by deionized water to 5 mL and was filtered through a 0.22 μm filter. Finally, the filtrate was tested by HPLC-MS/MS (SHIMADAZU, LC-30AD/LCMS-8050).

#### 3.2.2. Liquid Chromatographic Conditions

The chromatographic column was the Diol-HILIC-120 column (100 mm × 2.1 mm, 1.9 μm). Mobile phase A was 0.05% formic acid aqueous solution; mobile phase B was 0.1% formic acid acetonitrile. The flow rate was 0.3 mL/min. The column temperature was 40 °C. The injection volume was 5 μL.

For gradient elution, the mobile phase B was controlled as 90% 0 min, 90% 1 min, 90%~35% 1.1 min, 35% 7.0 min, 35%~60% 7.1 min, 60%~90% 10.0 min, and 90% 13.0 min.

#### 3.2.3. Mass Spectrometry Condition

The negative ion scanning of the electrospray ionization (ESI) source was selected, determined to be [M − H]^−^ as the precursor ion. The specific mass spectrum parameters were shown in Table 5, and the quantitative and qualitative ions and collision energy were shown in Table 2.

#### 3.2.4. Condition Optimization

In the pretreatment condition (as described in Section 3.2.1), the sample was precipitated by methanol and alcohol, the isoelectric point of the sample was adjusted with formic acid, and the macromolecular protein was removed by combining nitrogen blowing and concentration to achieve the purpose of sample purification. This process does not require using highly toxic chemical reagents, which is more environmentally friendly and safer.

In liquid chromatography (as described in Section 3.2.2), column types (C18, C18-AQ, and HILIC columns), mobile phase pH values, and initial proportions of mobile phase B (75%, 83%, 90%) were compared. The result showed that the optimal condition was optimal for the HILIC column, acidic mobile phase, and 90% initial proportion of mobile phase B.

In the mass spectrometry condition (as described in Section 3.2.3), the ESI source for positive and negative ion scanning was compared. The result showed that the [M − H]^−^, the parent ion, was optimal.

### 3.3. Effect of Heat Treatment Intensity on 3′-Sialyllactose Content in Edible Bird’s Nest

#### 3.3.1. Preparation of Samples with Different Heat Treatment Temperatures

The edible bird’s nest (dried) was crushed and weighed (1.00 g) in a 45 mL glass container. The sugar content was adjusted to 4%. Subsequently, the sample was heated to 90 °C within 6 min, kept for 30 min, and finally cooled to room temperature within 15 min. Similarly, samples with different heat treatment temperatures of 95 °C, 100 °C, 115 °C, 120 °C, and 128 °C were prepared.

#### 3.3.2. Preparation of Samples with Different Heat Treatment Times

The edible bird’s nest (dried) was crushed and weighed (1.00 g) in a 45 mL glass container. The sugar content was adjusted to 4%. Subsequently, the sample was heated to 121 °C within 6 min, kept for 10 min, and finally cooled to room temperature within 15 min. Similarly, samples with different heat treatment times of 0 min, 15 min, 20 min, 30 min, and 40 min were prepared.

### 3.4. Preparation of Samples with Different Sugar Contents

The edible bird’s nest (dried) was crushed and weighed (1.00 g) in a 45 mL glass container. The sugar (monocrystal rock sugar) content was adjusted to 2%. Subsequently, the sample was heated to 121 °C within 6 min, kept for 30 min, and finally cooled to room temperature within 15 min. Similarly, samples with different sugar contents of 4%, 6%, and 10% were prepared.

### 3.5. Method Validation

According to the Chinese standard (GB/T 27417-2017) [26], the LC–MS/MS procedure for quantifying 3′-sialyllactose was validated in terms of linearity, sensitivity, RSD, recovery, residue, etc.

### 3.6. Statistical Analysis

Results were expressed as mean ± standard deviations of triplicate analyses for each sample. The statistical analyses were performed using SPSS (version 23.0, SPSS Inc., Chicago, IL, USA). A comparison of the means was ascertained by Tukey’s test at a 5% level of significance using a one-way analysis of variance (ANOVA).

## 4. Conclusions

In this study, a high-performance liquid chromatography tandem mass spectrometry method was established for determining 3′-sialyllactose of edible bird’s nest. This method had the advantages of high sensitivity, good linear relationship, low detection limit, high precision, and accuracy. Compared with the existing methods, this method used methanol to pretreat the sample, which was simpler for sample preparation, and eliminated the possible interference caused by other reagents. It was suitable for the rapid detection of 3′-sialyllactose in edible bird’s nests and their products. Furthermore, the effects of the heat treatment process and sugar content on the 3′-sialyllactose content of the edible bird’s nest product were investigated. The results showed that within the tested range, the content of 3′-sialyllactose in edible bird’s nest products increased with the increase of heat treatment strength. When the heat treatment temperature was above 121 °C, the heat treatment time was above 30 min, and the sugar addition was 4%, it was more conducive to the formation of 3′-sialyllactose. In addition, the method established in this study was used to determine and analyze the 3′-sialyllactose content of edible bird’s nest products sold in the market. Compared with the 100 °C home-stewing dry-edible bird’s nest, the 3′-sialyllactose content in ready-to-eat edible bird’s nest products was higher, which is consistent with the conclusion that stronger heat treatment intensity can improve the nutritional value of edible bird’s nests. In short, this study provided a new method for detecting the nutritional value of edible bird’s nests, as well as a new direction for improving the nutritional value of edible bird’s nest products.

## Figures and Tables

**Table 1:** Comparison of liquid chromatography–mass spectrometry methods for 3′-sialyllactose.

NO.	Sample Type	Pretreatment Method	LOD (μg/kg)	RSD (%)	Rate of Recovery (%)	Reference
1	Breast milk	After centrifugation and alcohol precipitation, human milk was reduced with NaBH_4_, washed by graphite carbon column, and then dried in vacuum for detection.	8.3	/	60~100	[21]
2	Breast milk	The sample was refrigerated to remove the lipid layer, reduced with NaBH_4_, incubated in 65 ℃ water bath for 90 min, and then purified and extracted by non-porous graphized carbon solid-phase extraction.	/	<10	91.2~101.8	[22]
3	Milk	Cow milk was degreased by centrifugation and tested after deproteinization by methanol, acetonitrile, and ultrafiltration membrane.	5.0	<5	90~100	[23]
4	Rat plasma	Methanol was selected for protein precipitation in rat plasma samples. The methanol extract was totally evaporated with a vacuum concentrator and reconstituted with the solution comprising 10 mM ammonium acetate (pH 4.5) and acetonitrile (40:60, *v*/*v*).	/	<5	88.6~94.7	[19]
5	Edible bird’s nest	Bird’s nest samples were precipitated with 0.1% formic acid and methanol, and then dissolved in ultrapure water after vacuum concentration.	0.3	<5	91.6~98.4	This experiment

**Table 2:** Multi-reaction monitoring ion pairs and mass spectrometry conditions.

Analyte	Precursor Ion	Product Ion	Dwell Time, ms	Collision Energy	Interface Voltage, kV
3′-sialyllactose	632.15	290.15 *	197	28	−1.5
		142.10	197	40	−1.5

* is a quantitative ion.

**Table 3:** Recovery rate and relative standard deviation of 3′-sialyllactose in edible bird’s nest.

Analyte	Add Scalar Quantity, μg/kg	Recovery Rate, %	RSD, %
3′-sialyllactose	50	91.6	2.0
	100	96.8	1.5
	200	98.4	2.2

**Table 4:** Comparison of 3′-sialyllactose content in different edible bird’s nest products (*n* = 2, *p* < 0.05).

NO.	Type	Batch Number	Net Content, g	3′-Sialyllactose Content, μg/kg
1	Sample-1	202,209,189	70	6.60 ± 0.46 ^f^
2	Sample-2	20,221,207	45	73.84 ± 6.60 ^e^
3	Sample-3	UL16090	70	175.94 ± 6.26 ^d^
4	Sample-4	20,221,205	110	226.19 ± 4.36 ^c^
5	Sample-5	UK26041	138	261.34 ± 5.34 ^b^
6	Sample-6	UK04143	180	331.29 ± 6.91 ^a^
7	Sample-7	20,221,208	50	113.02 ± 5.31 ^d^
8	Sample-8	VK08154	70	166.14 ± 4.21 ^d^
9	Sample-9	XPVJ1608	100	305.22 ± 4.71 ^a^

The same letter means no significant difference at the 0.05 level, while different letters mean significant difference at the 0.05 level. The average value of the largest group is labeled as (a), and the groups with significant differences increase alphabetically according to the average size.

**Table 5:** Mass spectrometric parameters.

Parameters	Gas Flow (L·min^−1^)	Heating Gas Flow Rate (L·min^−1^)	Drying Gas Flow Rate (L·min^−1^)	Interface Temperature (°C)	DL Temperature (°C)	Heating Block Temperature (°C)	CID Gas (kPa)
Numerical value	3	10	10	300	250	400	270

## Data Availability

Not applicable.

## Sample Availability

Samples of the compounds are not available from the authors.
